# Cell Wall Biogenesis Protein Phosphatase CrSsd1 Is Required for Conidiation, Cell Wall Integrity, and Mycoparasitism in *Clonostachys rosea*

**DOI:** 10.3389/fmicb.2020.01640

**Published:** 2020-07-15

**Authors:** Binna Lv, Na Jiang, Rakibul Hasan, Yingying Chen, Manhong Sun, Shidong Li

**Affiliations:** Institute of Plant Protection, Chinese Academy of Agricultural Sciences, Beijing, China

**Keywords:** *Clonostachys rosea*, Ssd1, cell wall integrity, mycoparasitism, biocontrol efficacy, conidiation

## Abstract

Cell wall biogenesis protein phosphatases play important roles in various cellular processes in fungi. However, their functions in the widely distributed mycoparasitic fungus *Clonostachys rosea* remain unclear, as do their potential for controlling plant fungal diseases. Herein, the function of cell wall biogenesis protein phosphatase CrSsd1 in *C. rosea* 67-1 was investigated using gene disruption and complementation approaches. The gene-deficient mutant ΔCrSsd1 exhibited much lower conidiation, hyphal growth, mycoparasitic ability, and biocontrol efficacy than the wild-type (WT) strain, and it was more sensitive to sorbitol and Congo red. The results indicate that *CrSsd1* is involved in fungal conidiation, osmotic stress adaptation, cell wall integrity, and mycoparasitism in *C. rosea*.

## Introduction

Cell walls are essential structures that help organisms resist environmental stresses and protect a variety of natural cellular processes. In fungi, the biogenesis and integrity of cell walls are vital for fungal pathogenesis and survival (Scrimale et al., 2009; Wei et al., 2016), and their remodeling and expansion determine cell growth and reproduction (Fuchs and Mylonakis, 2009; Wang et al., 2013; Feng et al., 2017). Several cell wall building-related proteins have been identified in fungi, including the RNA-binding protein Ssd1 that was first cloned from *Saccharomyces cerevisiae* and is involved in various cellular processes and pathways such as cell wall integrity, signal transduction, and the cell cycle (Kaeberlein and Guarente, 2002; Reinke et al., 2004; Jansen et al., 2009). Ssd1 is highly conserved in fungi and contains a RNase II (RNB) domain (SMART No. SM00955), which is the catalytic domain of ribonuclease II, suggesting that it may be involved in post-transcriptional regulation by directly interacting with messenger RNAs (mRNAs; Mir et al., 2009; Kurischko et al., 2011b). The protein was also found to be remarkably enriched in message transmission-related proteins during cell wall biogenesis (Jansen et al., 2009).

In *S. cerevisiae*, Ssd1 regulates cell wall remodeling by inhibiting the translation of related proteins (Luukkonen and Séraphin, 1999; Wanless et al., 2014), and its inactivation is regulated by the nuclear Dbf2-related (Ndr)/large tumour suppressor (LATS) family protein kinase Cbk1 through phosphorylation of the N-terminal region of Ssd1 (Bidlingmaier et al., 2001; Du and Novick, 2002; Kurischko et al., 2011a). Lack of this regulation significantly impairs bud expansion and causes severe aberrant cell wall organization (Moriya and Isono, 1999). Three independent pathways separately mediated by Mpt5, Ssd1, and Pkc1 regulate cellular integrity; Mpt5 and Ssd1 act post-transcriptionally during cell wall biosynthesis and maintain cell structure as upstream regulators (Kaeberlein and Guarente, 2002), while Pkc1 activates a mitogen-activated protein kinase (MAPK) cascade that controls the transcription and expression of genes involved in cell wall formation (Gerik et al., 2005, 2008; Mir et al., 2009).

Orthologs of *Ssd1* have been characterized in several pathogenic fungi, and they exhibit various functions. In *Candida albicans*, *Ssd1* impedes the mutation of other genes and weakens the effects of mutations on cellular processes (Gank et al., 2008; Ohyama et al., 2010; Avrahami-Moyal et al., 2012). In *Colletotrichum orbiculare*, *Ssd1* is essential for penetration of appressoria into the epidermal cells of susceptible plants, and the *Ssd1*-deficient mutant displays enhanced basal resistance to *Nicotiana benthamiana* (Tanaka et al., 2007, 2009). Moreover, deletion of *Ssd1* in *Colletotrichum higginsianum* and *Magnaporthe grisea* leads to weakened penetration and virulence (Schmidpeter et al., 2017; Yu et al., 2019). However, the functions of orthologs of *Ssd1* in biocontrol fungi remain poorly understood.

*C. rosea* (syn. *Gliocladium roseum*) is a widely distributed mycoparasite associated with a range of pathogenic fungi, such as *Sclerotinia sclerotiorum*, *Rhizoctonia solani*, and *Botrytis cinerea* (Xue, 2003; Zhang et al., 2008; Kosawang et al., 2014). This species has great potential for controlling various plant fungal diseases and promoting crop growth (Atanasova et al., 2018), and functional genes in particular have attracted much attention. Fatema et al. (2018) demonstrated that two polyketide synthase genes, *PKS22* and *PKS29*, play important roles in the synthesis of antifungal agents, clonorosein A–D that are effective against *B. cinerea*. Dubey et al. (2016) found that the ABC transporter gene *ABCG29* is involved in fungal adaptation to oxidative stress in the early stages of mycelial development and biocontrol of *B. cinerea* and *Fusarium graminearum*. Sun et al. (2019a) indicated that the heat shock protein 70 gene, *crhsp*, had a remarkable effect on *C. rosea* morphological characteristics and significantly reduced its ability to parasitize *S. sclerotiorum* sclerotia. However, there have been no studies on the functions of *Ssd1*, and whether *Ssd1* is related to cell wall formation in *C. rosea* remains unknown.

In the present study, we identified and characterized the *CrSsd1* gene, which is orthologous to *S. cerevisiae Ssd1* and markedly upregulated during *C. rosea* parasitizing *S. sclerotiorum* (Sun et al., 2015b). Our results indicate that *CrSsd1* is involved in conidiation, responses to osmotic stress, cell wall integrity, and mycoparasitism in *C. rosea*. This knowledge reinforces our understanding of the mechanisms underlying *C. rosea* mycoparasitism and lays a foundation for developing new potent biocontrol agents. To the best of our knowledge, this is the first report of CrSsd1 as a mycoparasitism-associated protein involved in *C. rosea* against fungal plant pathogens.

## Materials and Methods

### Fungal Strains

*C. rosea* 67-1 (ACCC 39160) was originally isolated from a vegetable yard in Hainan Province, China, using the sclerotia-baiting method (Zhang et al., 2004). *S. sclerotiorum* Ss-H (ACCC 39161) was separated from sclerotia-infected soybean stems in a field in Heilongjiang Province, China. *B. cinerea* TC-B1 was isolated from infected tomato fruits in a greenhouse (Sun et al., 2015b). All strains were maintained at 4°C in the Biocontrol of Soilborne Diseases Lab of the Institute of Plant Protection, Chinese Academy of Agricultural Sciences.

### Bioinformatics Analysis

The DNA sequence of *CrSsd1* was obtained from the draft genome sequence of *C. rosea* 67-1. NCBI^1^ and UniProt^2^ were used for BLASTp analysis. Functional domains of *CrSsd1* were predicted using SMART^3^. The Clustal X program was used for amino acid alignments. The phylogenetic tree was constructed by MEGA 7.0 using the maximum likelihood method with 1,000 bootstrap replicates.

### Quantitative Reverse Transcription PCR of *CrSsd1*

Strain 67-1 genomic DNA was extracted using a Biospin Fungus Genomic DNA Extraction Kit (Bioer Technology Co. Ltd., Hangzhou, China) according to the manufacturer’s instructions. Plasmid DNA was isolated using a Plasmid Miniprep Purification Kit (BioDev Co., Beijing, China).

We analyzed the expression levels of *CrSsd1* in strain 67-1 during different stages of mycoparasitizing sclerotia. Strain 67-1 was incubated on potato dextrose agar (PDA) at 26°C for 10 days, spores were washed with sterile water and adjusted to 1 × 10^7^ spores/ml, and spore suspensions were smeared evenly on a PDA plate and covered with cellophane. Uniformly sized sclerotia were placed onto the surface of 67-1 plates evenly after culturing for 48 h, and *C. rosea* 67-1 mycelia were collected at 8, 24, and 48 h and placed immediately in liquid nitrogen. Each treatment included five replicates. Total RNA was extracted using TRIzol reagent (Invitrogen, CA, USA) according to the manufacturer’s instructions. RNase-free DNase I (Invitrogen) was used to eliminate DNA contamination. Reverse transcription was performed using a cDNA FastQuant RT Kit (Tiangen, Beijing, China). Gene expression was analyzed by quantitative reverse transcription PCR (qRT-PCR) using a Bio-Rad IQ 5 Real-Time System (Bio-Rad, CA, USA) and SYBR Premix Ex Taq (Takara, Dalian, China) with primers listed in Table 1. Elongation factor gene *EF1* (GenBank accession number: KP274074) was used as a reference gene to normalize gene expression in *C. rosea* 67-1 under sclerotia induction (Sun et al., 2015a,b), and mycelial samples without added sclerotia acted as a control. The relative expression levels of *CrSsd1* were calculated using the 2^−∆∆Ct^ method, and three replicates were included for each sample.

**Table 1:** Primers used in this study.

Primer No	Primer	Sequence (5'–3')*^a^* ^,^ *^b^*	Relevant characteristics
1	CrSsd1-uF	GGTCTTAAUCAGGGGAGCAGCAGTTGG	PCR primers to amplify the *CrSsd1* upstream fragment for construction of *CrSsd1* deletion mutants
2	CrSsd1-uR	GGCATTAAUGGGGAGGGGAAGATAGCTAG	
3	CrSsd1-dF	GGACTTAAUGCCTCACAATCCGCTCTCTA	PCR primers to amplify the *CrSsd1* downstream fragment for construction of *CrSsd1* deletion mutants
4	CrSsd1-dR	GGGTTTAAUAGCTGAGTGAGGGGTGATAT	
5	CrSsd1-in-F	GGTCAACCCATCCACCCTG	PCR primers for identification of *CrSsd1* deletion transformants
6	CrSsd1-in-R	GCTGCATTGGGTTGAGCTG	
7	CrSsd1-out-F	GCGAAACCCAATTCCCAGTT	PCR primers for identification of *CrSsd1* deletion transformants
8	CrSsd1-out-R	CACTCCGACTTTGCTTGACC	
9	CrSsd1-yz-F	GGCGGACCCCTAATGATGTA	PCR primers for identification of *CrSsd1* deletion transformants
10	CrSsd1-yz-F	TTGCCATCCGAACCTTCTTC	
11	HPH-F	TGGAGCTAGTGGAGGTCAACA	PCR primers for amplification of the hygromycin resistant gene *HPH*
12	HPH-R	CGGTCGGCATCTACTCTATTC	
13	CrSsd1-F	GTCGATGAAGTCTGGTCCCA	PCR primers for identification of *CrSsd1* expression levels in RT-PCR assays
14	CrSsd1-R	CGCTGATCTCTTCCTCCTCA	
15	CrSsd1-com-F	CCCCCGGGCTGCAGgaattcGTTGTGGTGATCGTTGGAGG	PCR primers to amplify full-length *CrSsd1* including 1,441 bp upstream and 505 bp downstream fragments for complementation of the *CrSsd1* deletion mutant
16	CrSsd1-com-R	TCGACGGTATCGATaagcttTGTTCGTCACTAGCCTTAGGG	
17	EF1-F	TCGATGTCGCTCCTGACT	PCR primers for amplification of the reference gene *EF1* in qRT-PCR and RT-PCR assays
18	EF1-R	AGCGTGACCGTTTATTTGA	
19	CrSsd1-RT-F	TGGCAAGGTTTCACTGAAGG	PCR primers for amplification of the *CrSsd1* gene in qRT-PCR assays
20	CrSsd1-RT-R	TGCTGCAACAAACGAAGAGG	

a Respective exogenous enzyme sites are indicated by lowercase letters in the sequence.

b Underlined sequences are homologous recombination sequences of the pKH-KO vector.

### Generation of Gene Deletion and Complementation Mutants

The plasmid pKH-KO containing two uracil-specific excision reagent (USER) cloning sites (USC1 and USC2) on either side of the hygromycin resistance gene *hph* was used to construct a *CrSsd1* disruption vector (Frandsen et al., 2008). Upstream and downstream flanking sequences of *CrSsd1* were amplified using primer pairs CrSsd1-uF/CrSsd1-uR and CrSsd1-dF/CrSsd1-dR, respectively, and cloned into two USC sites using the USER-friendly cloning method to generate *CrSsd1*-deletion vector pKH-KO-CrSsd1.

For construction of the gene complementation vector, the full-length sequence of *CrSsd1*, including the promoter, protein-coding, and terminator regions was amplified from 67-1 genomic DNA and cloned into the pKN vector (carrying the G418 resistance gene *neo*; Kong et al., 2019). The resulting gene deletion and complementation vectors were transformed into protoplasts of 67-1 and ΔCrSsd1, respectively, to generate gene deletion and complementation mutants using protoplast formation and transformation of *C. rosea* (Sun et al., 2017). Primers (Table 1) were designed and mutants were verified by PCR and DNA sequencing. Furthermore, the expression levels of *CrSsd1* in wild-type (WT), deletion and complementation strains were tested using reverse transcription PCR (RT-PCR) with primers CrSsd1-F and CrSsd1-R and reference gene *EF-1* gene (Table 1; Sun et al., 2015a,b).

### Fungal Growth, Conidiation, and Stress Tolerance

To analyze differences in vegetative growth among *C. rosea* 67-1, ΔCrSsd1, and ΔCrSsd1-C strains, agar blocks (3 mm) of strains were inoculated onto the center of a PDA plate and cultured at 26°C. The size and morphology of colonies were measured daily. After 15 days, fungal spores were collected by adding 5 ml sterile distilled water, and spores were counted under a BX41 microscope (Olympus, Tokyo, Japan). The conidial germination rates of all strains were determined. Spore suspensions of WT, ΔCrSsd1 and ΔCrSsd1-C strains with a concentration of 1 × 10^7^ spores/ml were prepared and inoculated in potato dextrose (PD) broth on a rotary shaker at a speed of 180 rpm. Samples were incubated at 26°C, and the germinated conidia were counted at 8 and 16 h post inoculation. To evaluate the stress response, cultures were grown on PDA plates amended with different stress agents [1 M NaCl, 1 M KCl, 1 M glycerin, 1 M sorbitol, 20 mM H_2_O_2_, 0.03% SDS, and 0.3 mg/ml Congo red (CR)] for 10 days. Diameters of colonies were counted, and microscopic observation of the hypha under different stress conditions was performed with a fluorescence microscope system (DM6 B, Leica, Germany). All assays were repeated three times.

### Antagonistic Activity Against *Botrytis cinerea*

Antagonistic activity of *C. rosea* 67-1, ΔCrSsd1, and ΔCrSsd1-C strains against *B. cinerea* was tested on 9 cm PDA plates. A 3-mm agar plug of strains was inoculated 2 cm from the edge of the plate and cultured at 26°C for 5 days. A plug of *B. cinerea* was then placed equidistant from the other side of the plate and cultured at 26°C for 20 days. The distance of hyphal extension for each strain was measured (Dubey et al., 2014; Tzelepis et al., 2015; Filizola et al., 2019).

### Mycoparasitic Ability Against *Sclerotinia sclerotiorum* Sclerotia

Sclerotia of uniform size were surface-sterilized with 1% NaClO for 3 min, rinsed three times with sterile water, and then immersed in spore suspensions of wild-type (WT) 67-1, ΔCrSsd1, and ΔCrSsd1-C strains at a concentration of 1 × 10^7^ spores/ml for 10 min. Sclerotia were picked and placed onto a piece of wet sterile filter paper in a Petri dish (diameter 9 cm) and incubated at 26°C. Treatment with sterile water was used as a control. The number of sclerotia infected by the transformants was counted under a stereo microscope (SMZ-10, Nikon, Tokyo, Japan) at 8, 16 and 24 h. Sclerotia covered with *C. roses* mycelia were regarded as parasitized, and parasitic rates of all strains were calculated (Sun et al., 2017). After 7 days, we investigated parasitic severity of sclerotia using a BX41 inverted microscope (Olympus) based on a four-grade scale (0 = no *C. rosea* hyphae were detected on the surface of sclerotia; 1 = loose *C. rosea* hyphae extended to the sclerotia; 2 = sclerotia were covered with *C. rosea* hyphae but not softened; and 3 = sclerotia were covered with *C. rosea* hyphae and exhibited soft rot; Sun et al., 2019a). A total of 30 sclerotia were tested for each treatment, and three replicates were performed.

### Control Efficacy Against Soybean Sclerotinia Rot

Pot experiments were carried out to test the ability of *C. rosea* 67-1, ΔCrSsd1, and ΔCrSsd1-C strains to control *S. sclerotiorum* on soybean in the greenhouse. Soybean seeds (Zhonghuang 13; Institute of Crop Sciences, CAAS, China) were sown in sterile soil in plastic pots (diameter 11 cm). When nine compound leaves had grown, seedlings were sprayed with 100 ml spore suspension (1 × 10^7^ spores/ml) from each strain. After drying for 2 h, an equivalent amount of *S. sclerotiorum* mycelial suspension was inoculated onto leaves. Plants treated with sterile water followed by the pathogen served as controls, and 12 pots were tested for each isolate. The greenhouse was maintained at 26–28°C and 60% relative humidity, and all pots were arranged randomly. After 7 days, disease severity of Sclerotinia rot was scored using grades 0–4 according to the percentage of lesions on soybean leaves (0 = no symptoms on soybean leaves; 1 = less than 10% lesions on soybean leaves, 2 = 10–30% lesions on soybean leaves; 3 = 30–50% lesions on soybean leaves; and 4 = over 50% lesions on soybean leaves). All unfolded compound leaves were checked and three replicates were performed for each treatment.

### Statistical Analysis

Statistical software SPSS 2.0 (Chicago, IL, USA) was used for ANOVA. Statistical tests were carried out using Tukey’s test for multiple comparisons and a *p* < 0.05 was considered statistically significant.

## Results

### Identification and Expression Levels of *CrSsd1*

**Figure 1:**
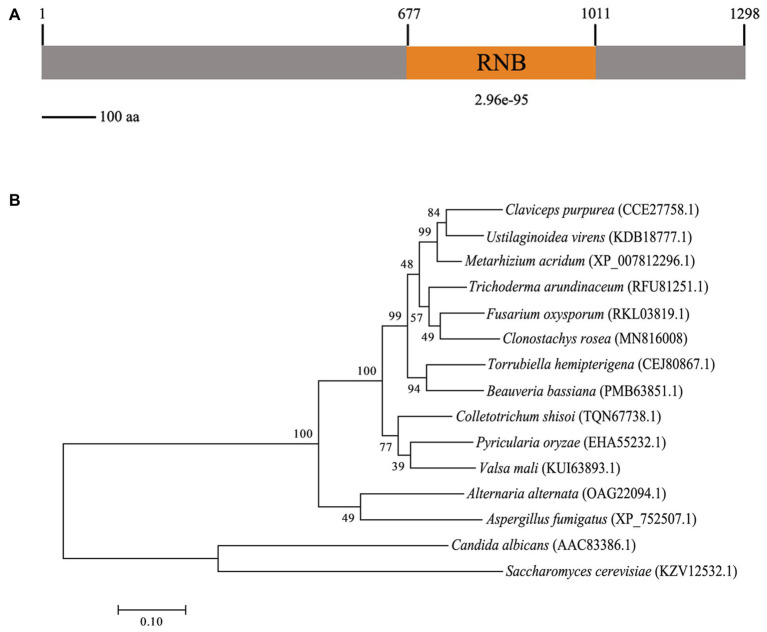
Characterization of the *Clonostachys rosea* CrSsd1 protein. **(A)** The domain structure of *C. rosea* CrSsd1 as annotated by SMART Mode (http://smart.embl.de/). **(B)** Phylogenetic analysis of CrSsd1 of *C. rosea* and its homologs from other fungi. Amino acid sequences were aligned by Clustal X and analyzed by MEGA 7.0 using the maximum likelihood method. Numbers in parentheses indicate GenBank accession numbers. Numbers at the nodes represent the bootstrap values of 1,000 bootstrap replicates. Bars = 0.10 and represent sequence divergence.

Gene cloning and bioinformatics analysis showed that *CrSsd1* (GenBank accession number: MN816008) is 3,894 bp in length with no introns and encodes a 1,298-amino-acid polypeptide that contains the RNB domain (Figure 1A), which is the catalytic domain of ribonuclease II. CrSsd1 shares 45.8% identity with *S. cerevisiae* Ssd1, which is involved in a range of cellular processes, including cell wall integrity, signal transduction, and RNA deterioration. Phylogenetic analysis and sequence alignment of *CrSsd1* with other fungal species revealed close homology with homologs of *Ssd1* in *Fusarium oxysporum* and *Trichoderma arundinaceum*, and it is highly conserved among various fungi (Figure 1B).

**Figure 2:**
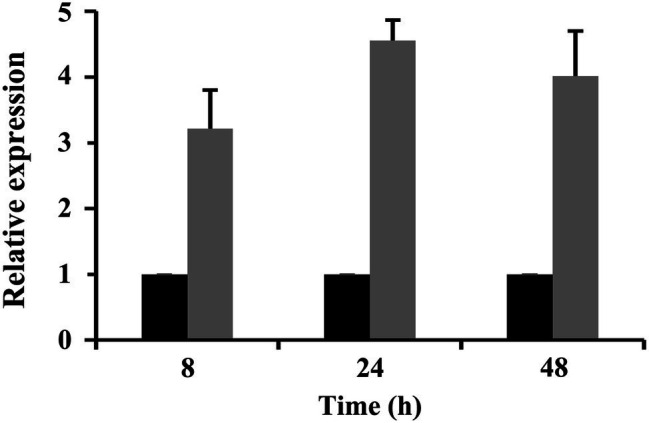
Expression levels of *CrSsd1* in *C. rosea* under sclerotia mycoparasitism conditions. Black columns represent samples added with fresh sclerotia. Gray columns represent controls (samples without sclerotia). The relative expression levels of *CrSsd1* were calculated using the 2^−∆∆Ct^ method. Error bars show the standard deviation (SD) of three replicates.

The expression levels of *CrSsd1* in 67-1 were also investigated during different stages of mycoparasitizing sclerotia by qRT-PCR. Analysis of gene expression indicated that *CrSsd1* was upregulated in *C. rosea* throughout mycoparasitism, particularly at 24 h, and expression levels were more than four-fold higher than the control (Figure 2), which is consistent with the transcriptome data from *C. rosea* parasitizing *S. sclorotiorum* (Sun et al., 2015b).

### *CrSsd1* Disruption and Complementation

**Figure 3:**
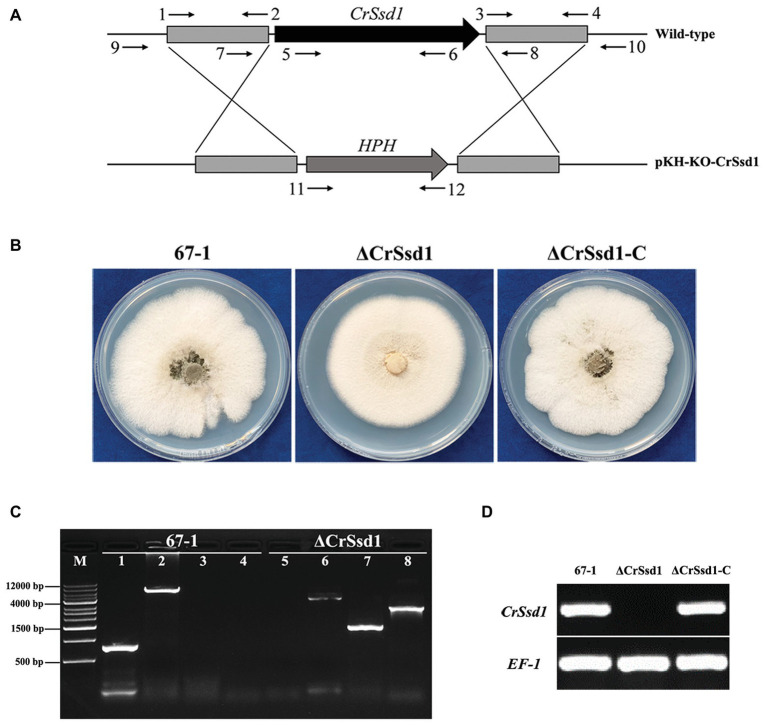
Construction of replacement vectors and confirmation of *CrSsd1* deletion mutants. **(A)** Schematic representation of the gene disruption strategy. The hygromycin resistance cassette (*hph*) was cloned into the corresponding sites of vector pKH-KO-CrSsd1 to replace the 3,894 bp *CrSsd1* open reading frame. The annealing sites of primers are indicated by small black arrows. **(B)** Colony morphologies of 67-1, ΔCrSsd1, and ΔCrSsd1-C grown on potato dextrose agar (PDA) plates at 26°C for 10 days. **(C)** PCR analysis of 67-1 and CrSsd1 deletion mutant strains using primers 5/6 (CrSsd1-in-F/R), 9/10 (CrSsd1-yz-F/R), 11/12 (HPH-F/R), and 9/12 (CrSsd1-yz-F/HPH-R). Lanes 1–4, PCR products amplified with the above primers using 67-1 as template; lanes 5–8, PCR products amplified with the above primers using ΔCrSsd1 as template. Primer numbers refer to binding sites shown in Table 1. **(D)** Reverse transcription PCR (RT-PCR) analysis of *CrSsd1* gene expressions in 67-1, ΔCrSsd1, and ΔCrSsd1-C strains, using *CrSsd1* specific CrSsd1-F/R primers (Table 1). The RT-PCR product of 247 bp was expected from 67-1 and ΔCrSsd1-C but not in the *CrSsd1* deletion mutants.

To identify the role of *CrSsd1* in *C. rosea*, single gene deletion mutants were generated using a homologous recombination strategy (Figure 3A). Among 187 hygromycin-resistant transformants, three ΔCrSsd1 strains with identical phenotypic characteristics were confirmed by PCR analysis with primers CrSsd1-in-F/R (inside of the target gene), CrSsd1-yz-F/R (outside of the homologous fragment), HPH-F/R (on both ends of the *hph* gene), and CrSsd1-yz-F/HPH-R (Figure 3C). Moreover, fragments amplified by primer pair CrSsd1-yz-F/R were sequenced, and the results showed that the *CrSsd1* gene was successfully replaced with a hygromycin B resistance cassette as expected. For complementation of *CrSsd1*, the vector pKN-CrSsd1-C was transformed into the ΔCrSsd1 strain and 11 complementation strains were finally obtained. RT-PCR verification demonstrated a complete loss of *CrSsd1* transcript in ΔCrSsd1 mutants, whereas specific products were detected in the WT and complementation strains. In addition, the expression of *EF-1* gene was detected in all strains (Figure 3D).

### Effects of *CrSsd1* on Fungal Growth, Conidiation and Sensitivity to Stresses

**Figure 4:**
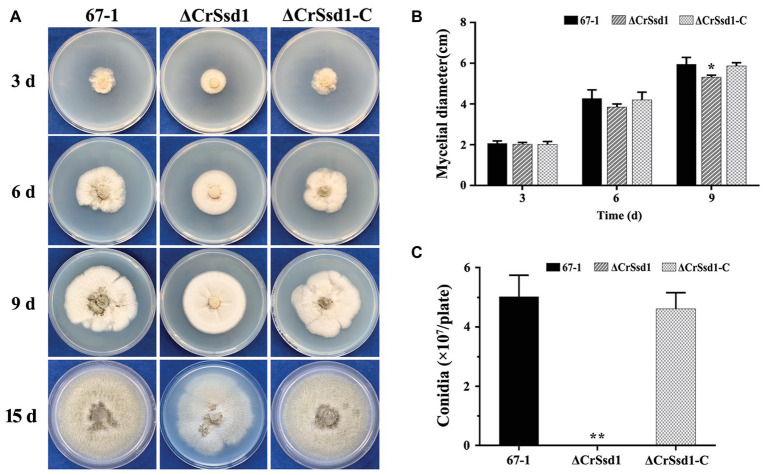
Impact of *CrSsd1* deletion on mycelial growth and conidia formation. **(A)** Mycelial growth of 67-1, ΔCrSsd1, and ΔCrSsd1-C on PDA medium after 3, 6, and 9 days of incubation, and conidiation of these strains on PDA after 15 days. **(B)** Statistical analysis of colony diameters in **(A)**. **(C)** Number of conidia produced by each strain on PDA plates. The data are the means of the three ΔCrSsd1 and ΔCrSsd1-C mutants and the means and standard errors are calculated from three independent replicates. Statistical tests were carried out using Tukey’s test for multiple comparisons. Asterisks indicate statistically significant differences (*p* < 0.05).

Three ΔCrSsd1 and ΔCrSsd1-C mutants were selected to analyze the functions of *CrSsd1* gene. The colony morphology showed that ΔCrSsd1 mutants had flatter and thinner mycelia than those of the WT 67-1 and the complemented transformant ΔCrSsd1-C (Figure 3B). Moreover, the mycelial growth rates of mutants were slightly slower than that of the WT and ΔCrSsd1-C strains. When grown on PDA for 9 days, the colony diameter of 67-1 reached 5.95 cm, while that of ΔCrSsd1 was 5.29 cm, and the difference was significant (*p* < 0.05; Figures 4A,B). Surprisingly, gene-deficient strains lost almost all ability to undergo conidiation. After incubation on PDA for 15 days, only 1 × 10^6^ spores/plate were harvested for ΔCrSsd1, compared with 4.9 × 10^7^ spores/plate for the WT strain (*p* < 0.01; Figures 4A,C). Conidial germination rate of the ΔCrSsd1 mutants was 46.9%, significantly lower than that of the WT strain (68.7%) at 8 h (*p* < 0.05); however, both strains increased to approximately 100% at 16 h (Supplementary Figure S1). The complemented transformants showed similar results with WT.

**Figure 5:**
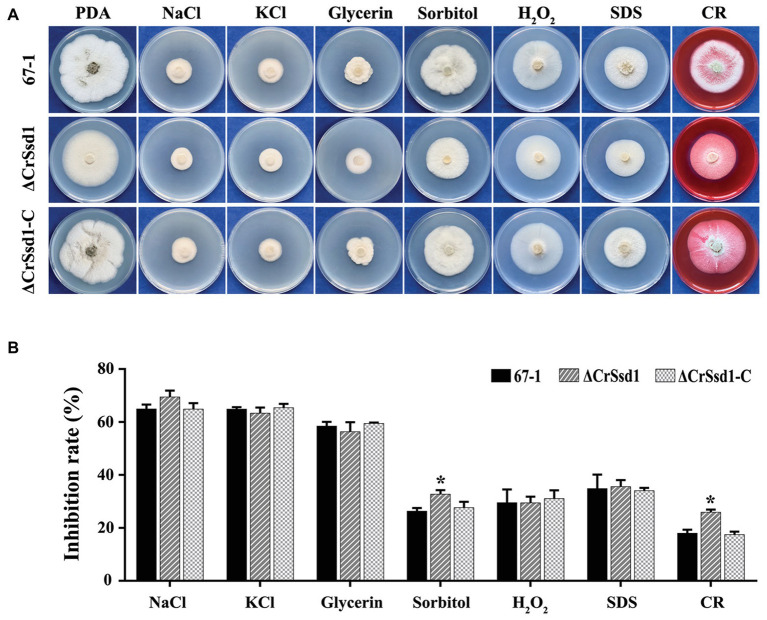
Sensitivity of 67-1, ΔCrSsd1, and ΔCrSsd1-C to diverse stresses. **(A)** Sensitivity of strains grown on PDA plates containing different stress agents; 1 M NaCl, 1 M KCl, 1 M glycerin, 1 M sorbitol, 20 mM H_2_O_2_, 0.03% SDS, and 0.3 mg/ml Congo red (CR). Images were captured after 10 days of incubation at 26°C. **(B)** Inhibition of mycelial growth compared with non-treated controls. The data are the means of three mutants, and the means and standard errors are calculated from three independent replicates. Statistical tests were carried out using Tukey’s test for multiple comparisons. Asterisks indicate statistically significant differences (*p* < 0.05).

The sensitivity of mutants to a variety of environmental stresses, including osmotic stress, oxidative stress, cell membrane stress, and cell wall stress, was investigated. The results showed no significant differences among strains under treatment with NaCl (1 M), KCl (1 M), glycerin (1 M), H_2_O_2_ (20 mM), or SDS (0.03%). However, interestingly, ΔCrSsd1 grew much slower in media containing sorbitol (1 M) or CR (0.3 mg/ml) compared with WT and complemented strains, indicating that ΔCrSsd1 deletion mutants were more sensitive to osmotic and cell wall stresses (Figures 5A,B). To further investigate the stress sensitivity of ΔCrSsd1 and 67-1, the hyphal phenotypes under different stress conditions were observed. Our findings demonstrated that the loss of *CrSsd1* impaired hyphae branching under NaCl, KCl, sorbitol, and CR, indicating that the *CrSsd1* gene played an important role in *C. rosea* response to osmotic and cell wall stresses (Supplementary Figure S2).

### Effects of *CrSsd1* on Antagonistic Activity, Mycoparasitism and Control Efficacy

**Figure 6:**
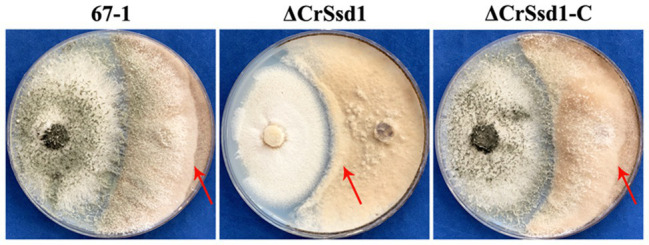
Impact of *CrSsd1* deletion on the antagonistic activity of *C. rosea*. Plate confrontation assay of 67-1, ΔCrSsd1, and ΔCrSsd1-C against *Botrytis cinerea* at 20 days post-inoculation. Red arrows indicate the hyphal extension distance of each strain toward *B. cinerea*.

*In vitro* antagonistic activity tests showed that *C. rosea* 67-1, ΔCrSsd1, and ΔCrSsd1-C strains could all overgrow a colony of *B. cinerea* after culturing for 20 days. However, the hyphal extension ability was decreased by 41.3% for ΔCrSsd1 mutants compared with the WT strain (*p* < 0.05), and the complemented strain ΔCrSsd1-C recovered this ability almost to the WT level (a decrease of only 3.6%; Figure 6).

**Figure 7:**
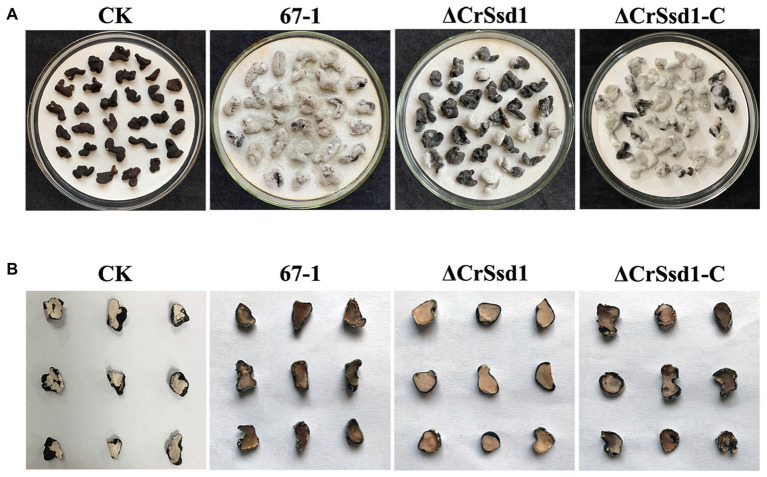
Mycoparasitism of *C. rosea* strains against *Sclerotinia sclerotiorum* sclerotia. **(A)** External phenotypes of healthy and infected sclerotia. **(B)** Transection of infected and uninfected sclerotia. Images were captured after 7 days incubation at 26°C.

No hyphae of ΔCrSsd1 mutants were detected on the surface of the sclerotia at 8 h after inoculation, and the parasitic rate was 20.5% at 16 h, which was remarkably lower than the WT (57.8%, *p* < 0.05). By 24 h, hypha of 67-1 and ΔCrSsd1-C covered the whole sclerotia surface, while only 48.3% were parasitized by the ΔCrSsd1 mutants (Table 2). After 7 days of cultivation in a moist environment, the mycoparasitism level of ΔCrSsd1 on sclerotia was markedly reduced compared with that of WT 67-1 and complemented strain ΔCrSsd1-C. From the external phenotype and the inner structure of the sclerotia, we could see that infected sclerotia were completely softened and rotten, resulting in high parasitic severity (grade 4), whereas those treated with the ΔCrSsd1 deletion mutant were covered only sparsely in hyphae and remained relatively firm, equating to mycoparasitism grade 1, indicating that deletion of the *CrSsd1* gene substantially weakened the mycoparasitism of *C. rosea*. Additionally, mycoparasitic ability was recovered in the complemented strain (Figures 7A,B).

**Table 2:** Parasitic rate of *C. rosea* strains against *S. sclerotiorum* sclerotia.

Strain	8 h (%)	16 h (%)	24 h (%)
WT	4.3 ± 0.5 a	57.8 ± 0.6 a	100.0 ± 0.0 a
ΔCrSsd1	0.0 ± 0.0 b	20.5 ± 0.9 b	48.3 ± 1.3 b
ΔCrSsd1-C	4.1 ± 0.9 a	55.3 ± 1.0 a	100.0 ± 0.0 a

Data are the means ± SD of three replicates of three mutants. Different letters in a column indicate significant differences according to Tukey’s test (*p* < 0.05).

**Figure 8:**
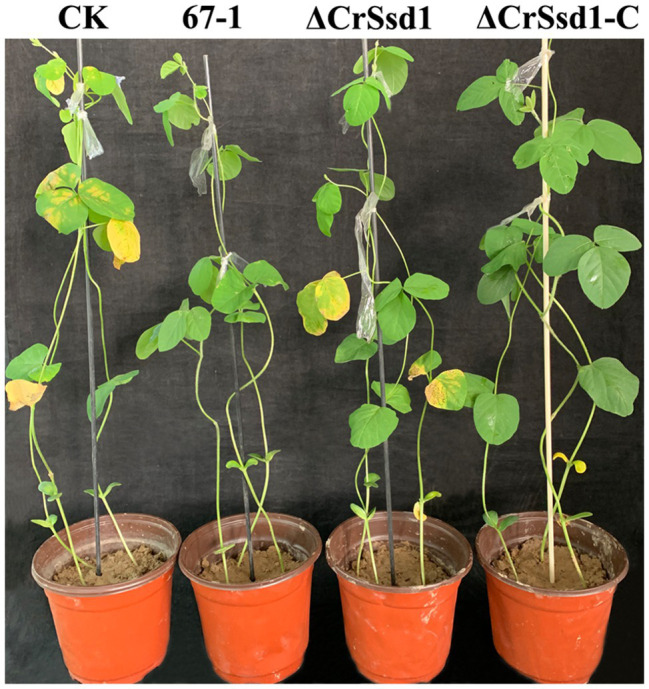
Impact of *CrSsd1* deletion on efficacy against soybean Sclerotinia rot. Soybean Sclerotinia rot by *C. rosea* 67-1, ΔCrSsd1, and ΔCrSsd1-C after 7 days in a greenhouse. Plants treated with sterile water followed by the *S. sclerotiorum* pathogen served as controls (CK), and 12 pots were tested for each isolate. Disease severity of Sclerotinia rot was investigated after 7 days.

After inoculation with *S. sclerotiorum* for 7 days, severe leaf lesions were observed in control soybean seedlings. However, soybean seedlings treated with the biocontrol fungus 67-1 were much healthier and displayed less damage, consistent with excellent control efficacy against soybean Sclerotinia rot. Interestingly, when the *CrSsd1* gene was deleted, the control efficacy of the mutant was markedly reduced, while the efficiency was regained in the complemented strain (Figure 8 and Table 3), demonstrating that *CrSsd1* could dramatically affect the biocontrol efficacy of *C. rosea*.

**Table 3:** Control efficacy of *C. rosea* against soybean Sclerotinia stem rot.

Strain	Disease index	Control efficacy (%)
CK	62.8 ± 1.3 a	-
WT	21.3 ± 1.1 c	66.1 ± 1.2 a
ΔCrSsd1	45.9 ± 0.9 b	26.9 ± 1.1 b
ΔCrSsd1-C	24.7 ± 0.7 c	60.7 ± 1.5 a

Data are the means ± SD of three replicates of three mutants. Different letters in a column indicate significant differences according to Tukey’s test (*p* < 0.05).

## Discussion

The fungal cell wall is the first line of defense for protecting against environmental stresses, and any action to remodel it is tightly controlled to maintain balance with stress resistance and osmotic stability. Ssd1 is known to be involved in cell wall integrity and biosynthesis in several pathogenic fungi. To explore the functions of the cell wall biogenesis protein phosphatase CrSsd1 in mycoparasites, we investigated *CrSsd1* in *C. rosea* using gene deletion and complementation methods and found that loss of *CrSsd1* led to defects in conidiation, stress responses, mycoparasitism, and biocontrol efficacy. To the best of our knowledge, this is the first demonstration that *CrSsd1* plays an important role in conidiation and mycoparasitism in *C. rosea*, which is of great value for the development of biocontrol fungal agents.

Normal mycelial growth is crucial to ensure fungal vitality and other activities (Cota et al., 2008; Liu et al., 2016). In our current study, ΔCrSsd1 deletion mutants exhibited reduced hyphal growth, suggesting diverse functions for *CrSsd1* in different fungal pathogens. The ΔCrSsd1 strain also showed a significant reduction in conidiation, which plays important roles in the completion of the fungal life cycle, and the ability to produce conidia is essential for successful colonization and mycoparasitism (Iqbal et al., 2019; Kong et al., 2019; Sun et al., 2019b). These phenotypes indicate that *CrSsd1* is required for vegetative growth and asexual reproduction. The exploration of *CrSsd1* regulating fungal sporulation will be very interesting and worth to be further investigated.

Fungi respond in various ways to exogenous stresses in order to maintain cell shape and normal physiological processes (Leng and Zhong, 2015; Wang et al., 2019). The fungal cell wall is a highly dynamic structure and is the first barrier that interacts with diverse environmental stresses. In addition, cell well integrity is vital for survival and pathogenesis but the regulatory mechanisms are complicated (Reinke et al., 2004). In the present study, *CrSsd1* deletion mutants displayed greater sensitivity to the cell wall inhibitor CR, consistent with previous observations for *S. cerevisiae* and *C. albicans* (Moriya and Isono, 1999; Gank et al., 2008; Yanamoto et al., 2011). Ssd1 has been implicated in the maintenance of cell wall integrity in *C. albicans*, and deletion of *Ssd1* can render cells more susceptible to cell wall-perturbing agents such as Calcofluor white (Ram and Klis, 2006; Gank et al., 2008). Additionally, the loss of *CrSsd1* leads to increased sensitivity to sorbitol and osmotic stress agent, and overexpression of *Ssd1* rescues the sorbitol dependence of cell wall-defective mutants in *C. albicans* (Avrahami-Moyal et al., 2012). It was also noticed that the hyphal branching of the ΔCrSsd1 strains markedly lessened under the stresses of NaCl, KCl, sorbitol, and CR, which might be a possible explanation for *CrSsd1* regulating *C. rosea* response to osmotic and cell wall stresses. From these observations, we deduced that *CrSsd1* may perform a different regulatory mechanism in stress responses in different fungi. Nevertheless, until recently, there was no experimental evidence for the contribution of *CrSsd1* to cell wall integrity in mycoparasites.

During mycoparasitism, a host fungus is parasitized by and provides a nutrient source for another biocontrol fungus, such as species of the genus *Trichoderma*, and *C. rosea* (Karlsson et al., 2017; Nygren et al., 2018). Mycoparasitism comprises several steps; when encountering a fungal host, mycoparasites trigger gene expression associated with recognition, penetration, and parasitism, through various mechanisms related to mycoparasitism, antifungal activity, competition, and production of cell wall-degrading enzymes (Qualhato et al., 2013; Lysoe et al., 2017; Gomez-Rodriguez et al., 2018; Ramirez-Valdespino et al., 2018). Our findings confirmed that the deletion of *CrSsd1* severely impaired *C. rosea* antagonistic activity and mycoparasitic ability to *S. sclerotiorum* and *B. cinerea* and dramatically decreased the control efficacy against soybean Sclerotinia rot. These observations were further supported by analysis of *CrSsd1* gene expression during different stages of *C. rosea* parasitizing *S. sclerotiorum* sclerotia, which showed that *CrSsd1* was highly expressed throughout mycoparasitism, particularly in the first stage of infection. It has been reported that Ssd1 is an important component of the regulation of Ace2p activity and morphogenesis (RAM) pathway comprised of two kinases and four associated proteins and a conserved Cbk1 target involving phosphorylation in *S. cerevisiae* (Bidlingmaier et al., 2001). Current researches suggest that Cbk1 and RAM regulate polarized growth, mating efficiency, and cell wall morphogenesis (Schmidpeter et al., 2017). We hypothesize that *CrSsd1* influences the mycoparasitic activity and cell wall integrity of *C. rosea* by regulating the expressions of genes related to RAM pathway.

Herein, we analyzed the functions of *CrSsd1* in hyphal growth, conidiation, and stress responses in *C. rosea* and found that it is involved in cell wall integrity and osmotic stress. Additionally, we found that *CrSsd1* is involved in mycoparasitism and biocontrol efficacy. The results provide new insight into the mycoparasitism-associated mechanisms of *C. rosea* and may assist the development of new biocontrol agents for controlling fungal plant pathogens. In-depth studies will be needed to further clarify the exact regulatory mechanism, such as comparative analysis of transcription profiles.

## Conclusion

In summary, the CrSsd1 protein of *C. rosea* was demonstrated to be essential for conidiation and responses to sorbitol and CR. Furthermore, CrSsd1 was found to be involved in mycoparasitism and biocontrol efficacy, indicating that it plays diverse and essential roles in this fungus.

## Data Availability Statement

The datasets generated for this study can be found in the GenBank accession number: MN816008.

## Author Contributions

MS, SL, and BL conceived and designed the study. BL performed the experiments, analyzed the data, and wrote the manuscript. NJ, RH, and YC prepared the figures and tables. MS and SL provided funding and reviewed the manuscript. All authors contributed to the article and approved the submitted version.

## Conflict of Interest

The authors declare that the research was conducted in the absence of any commercial or financial relationships that could be construed as a potential conflict of interest.

## Supplementary Material

The Supplementary Material for this article can be found online at: https://www.frontiersin.org/articles/10.3389/fmicb.2020.01640/full#supplementary-material.

**Supplementary FIGURE S1**Impact of CrSsd1 deletion on conidial germination. Spore suspensions of WT, ΔCrSsd1, and ΔCrSsd1-C strains were inoculated in PD broth at 26°C, and the germinated conidia were counted at 8 and 16 h. The data are the means of three mutants, and the means and standard errors are calculated from three independent replicates. Statistical tests were carried out using Tukey’s test for multiple comparisons. Asterisks indicate statistically significant differences (*p* < 0.05).

**Supplementary FIGURE S2**Impact of CrSsd1 deletion on hyphae branching under diverse stresses. Sensitivity of 67-1, ΔCrSsd1, and ΔCrSsd1-C strains was determined on PDA plates containing different stress agents, 1 M NaCl, 1 M KCl, 1 M glycerin, 1 M sorbitol, 20 mM H_2_O_2_, 0.03% SDS, and 0.3 mg/ml Congo red (CR). Microscopic photos of the hypha under stresses were captured after 10 days of incubation at 26°C with a fluorescence microscope system.

## Footnotes

^1^http://www.ncbi.nlm.nih.gov/

^2^http://www.uniprot.org/blast/

^3^http://smart.embl.de/
