# Supplementary figures and images for: Cell Wall Biogenesis Protein Phosphatase CrSsd1 Is Required for Conidiation, Cell Wall Integrity, and Mycoparasitism in Clonostachys rosea
Source: Front Microbiol. 2020 Jul 15;11:1640. doi: 10.3389/fmicb.2020.01640 (PMC7373758; doi:10.3389/fmicb.2020.01640)

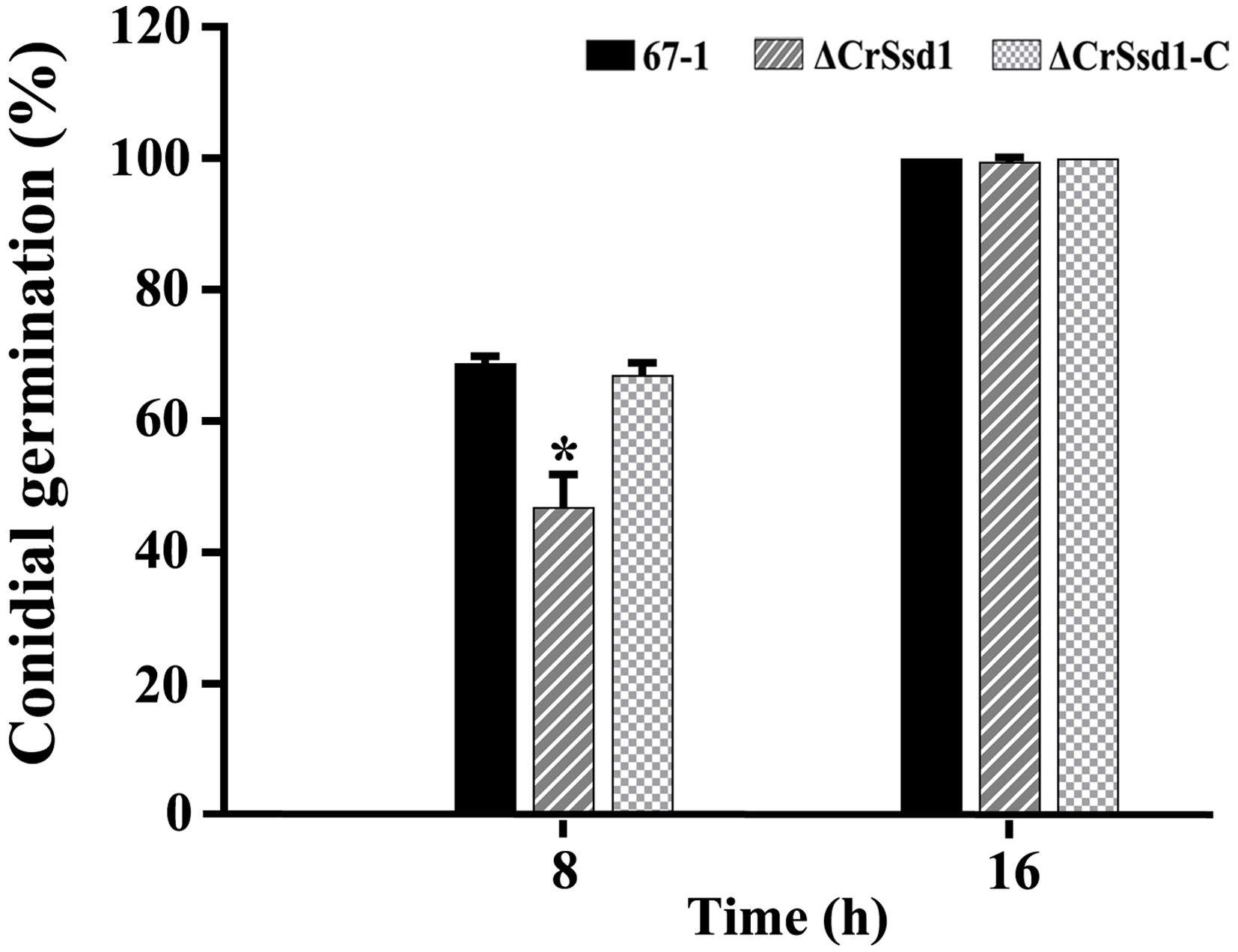

Supplement: Supplementary file 1 [file IMAGE_1.TIF]

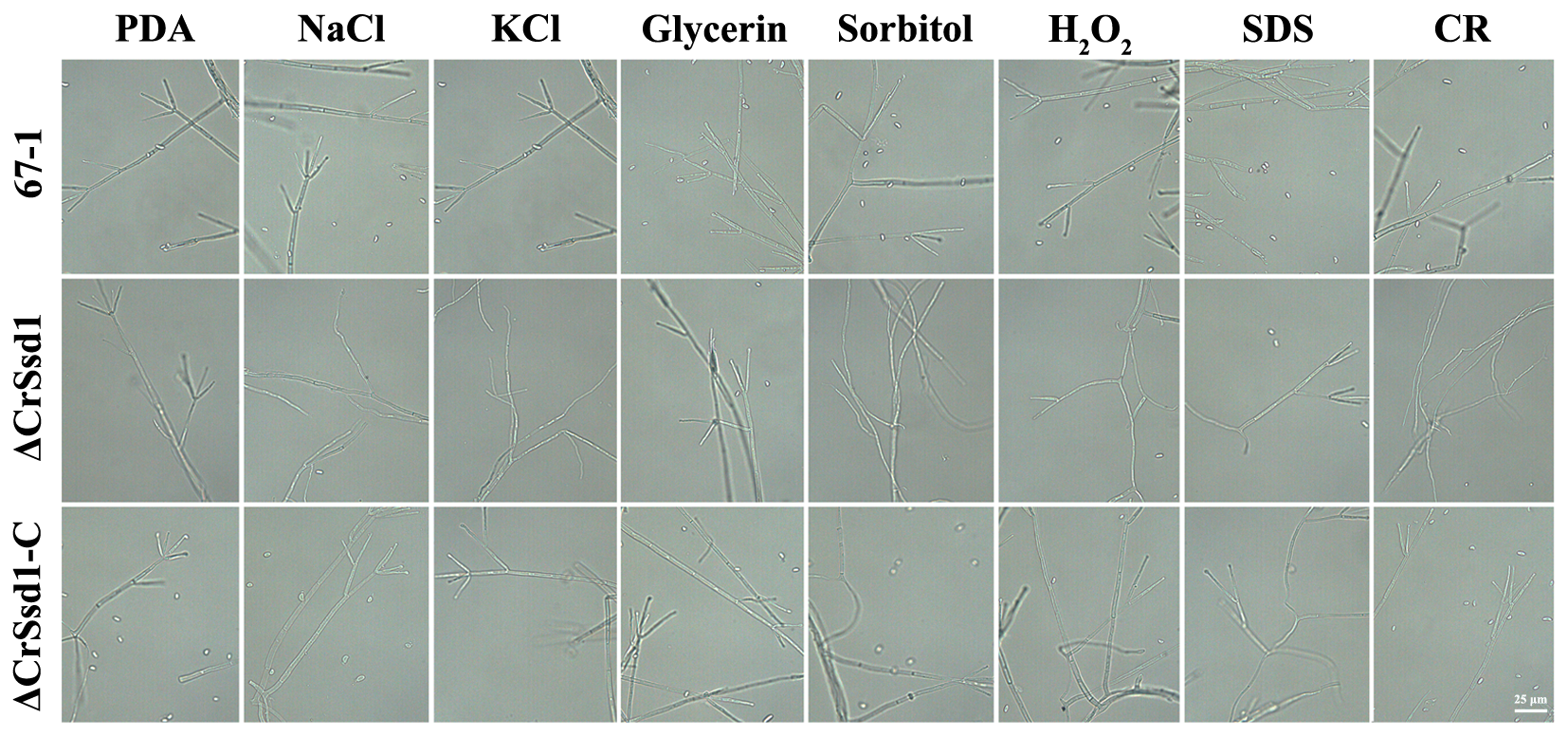

Supplement: Supplementary file 2 [file IMAGE_2.TIF]
